# Emergence of Postharvest Strawberry Fruit Rot Caused by *Penicillium citrinum* in China and Its Whole-Genome Sequencing

**DOI:** 10.3390/jof12040288

**Published:** 2026-04-17

**Authors:** Haohao Yan, Lili Jiang, Tianyu Guo, Mikael Motelica-Heino, Chong Wu

**Affiliations:** 1 Shandong Institute of Pomology, Tai’an 271000, China; 2 College of Horticultural Science and Engineering, Shandong Agricultural University, Tai’an 271018, China; 3 Institute of Earth Sciences of Orleans, University of Orléans, 45071 Orléans, France

**Keywords:** strawberry, postharvest fruit rot, *Penicillium citrinum*, whole-genome sequencing

## Abstract

China has the largest strawberry cultivation area worldwide and produces substantial quantities of the fruit. However, postharvest diseases of strawberries occur frequently, limiting their safe storage. In November 2025, a localized occurrence of postharvest fruit rot affecting strawberry (cv. Hongyan) was observed in Tai’an, China. A pathogenic fungus, designated CM-RB5, was isolated from diseased fruits and identified as *Penicillium citrinum* based on morphological characteristics and molecular analyses. This is the first report of *P. citrinum* causing postharvest fruit rot in strawberry. The genome of the pathogenic fungal strain CM-RB5 was sequenced using the Illumina MiSeq II and PacBio RS III platforms. Genome assembly analysis revealed the total sequence length of *P. citrinum* CM-RB5 to be 32,053,718 bp, with a GC content of 46.41%. Additionally, *P. citrinum* CM-RB5 was found to produce ochratoxin and citrinin. These findings provide insights that may facilitate the development of effective control strategies for postharvest fruit rot in strawberry, thereby ensuring the consumption of safe, high-quality fruit and strawberry-derived products.

## 1. Introduction

Strawberry (*Fragaria* × *ananassa* Duch.) is a non-climacteric fruit [1,2], rich in bioactive compounds such as polyphenols, anthocyanins, vitamins, and folic acid [3,4,5]. Strawberries have also been associated with various health-promoting properties, including anti-inflammatory, antioxidant, and detoxifying effects, as well as roles in supporting digestive and circulatory functions [6,7]. Strawberries are highly valued for their flavour, texture, and nutritional quality [8], and meet consumer demand for fresh fruits during off-season periods in northern regions of China. Consequently, they are widely appreciated by consumers and are also often referred to as the “queen of berries” [9,10]. Strawberries are among the most widely cultivated fruit crops in the world [11] and represent one of the most economically important berry crops, with production reaching 9.6 million tonnes in 2022 [12,13]. Major production regions include China, the United States of America, Egypt, Turkey, and Mexico [14], with China being the largest producer and possessing the largest cultivation area worldwide [15,16]. Owing to their short growth cycle, rapid economic returns, and high market value, strawberries are extensively processed into various products, including beverages, jams, dried fruits, and canned goods [17].

However, the high water content of strawberries (approximately 90%) renders them particularly susceptible to microbial damage, resulting in significant postharvest and economic losses [18,19,20,21]. Postharvest decay rates typically range from 30% to 50%, and shelf life is generally limited to less than 7 days, thereby significantly restricting the distribution and commercial value of the fruit [22,23]. The principal postharvest pathogens affecting strawberry fruit include species of *Botrytis*, *Alternaria*, *Fusarium*, *Mucor*, *Neopestalotiopsis*, and *Colletotrichum*, among others, which may pose potential risks to food safety [24,25,26,27]. These issues impede market expansion and the sustainable development of the strawberry industry [28], as well as contribute to consumer concerns about product safety [29]. Given the influence of diverse environmental factors, further isolation and identification of the microorganisms responsible for postharvest strawberry fruit rot are required.

Advances in sequencing technologies have enabled whole-genome sequencing to generate comprehensive genomic information about pathogens, including gene content, as well as genes encoding secondary metabolites and plant cell wall-degrading enzymes [30]. Genomic approaches are important tools for studying plant pathogenic fungi. Genome assembly and annotation facilitate the identification of mating-type loci and regulatory genes associated with pathogenicity and development, establishing a foundation for further work such as investigating the genetic diversity of pathogen populations, monitoring population drug resistance, and developing specific molecular diagnostic methods [30,31,32].

In November 2025, a localized occurrence of postharvest fruit rot affecting strawberry cv. Hongyan was observed in Tai’an, China (117.14° E, 36.18° N). The primary objective of this study was to isolate and identify the pathogen responsible for this disease through fungal morphological and molecular characterisation and to elucidate its genomic features. Furthermore, this work aims to provide a basis for future studies on disease management strategies, thereby supporting the development of effective biological and chemical control of this emerging postharvest disease.

## 2. Materials and Methods

### 2.1. Sample Collection and Fungal Isolation

In November 2025, approximately 30% disease incidence of 20 kg of strawberry fruit (cv. Hongyan) observed in Tai’an, China, showed postharvest fruit rot symptoms, leading to whole-fruit decay. Small segments (1–2 mm) of tissue were excised from symptomatic areas of three randomly selected fruit. These samples were surface-sterilised with 75% ethanol for 30 s and 5% sodium hypochlorite (NaOCl) for 3 min, rinsed three times with sterile distilled water, dried using sterile paper towels, and plated onto 9-cm Petri dishes containing potato dextrose agar (PDA) [33,34].

### 2.2. Morphological Identification

Three purified isolates were cultured on PDA plates at 25 °C for 7–11 days in the dark to assess colony morphology [33]. The conidial morphology was examined using an upright microscope (BX53F, Olympus, Tokyo, Japan).

### 2.3. PCR Amplification

Genomic DNA was extracted from fungal isolates using the Ezup Column Fungi Genomic DNA Purification Kit (Sangon Biotech, Shanghai, China). The internal transcribed spacer (ITS) region was amplified using the primer pair ITS1/ITS2 [35]. The amplification products were purified and sequenced by Sangon Biotech, Shanghai, China. Phylogenetic analyses of representative isolates were conducted with PhyloSuite v2, and trees were constructed using the MrBayes algorithm [36].

### 2.4. Pathogenicity Assays

Ten healthy strawberry (cv. Hongyan) fruits were surface-sterilised using 75% ethanol and rinsed with sterile distilled water. Five fruits were inoculated with conidial suspension (10^6^ spores/mL, determined by hemocytometer) of the isolate, while five fruits treated with sterile distilled water served as controls. The fruits were incubated at 25 °C and 75% relative humidity and the experiment was repeated twice. Following symptom development, the pathogen was re-isolated from lesions of inoculated strawberry fruit, thereby fulfilling Koch’s postulates [33].

### 2.5. Whole-Genome Sequencing

Whole-genome sequencing of strain CM-RB5 was performed to characterise the pathogen at the genomic level. Fungal mycelia of CM-RB5 were cultured in potato dextrose broth (PDB) with shaking for 72 h prior to DNA extraction. Genome sequencing was performed using the Illumina MiSeq II and PacBio RS III platforms (Shanghai Personal Biotechnology Co., Ltd., Shanghai, China). Functional annotation of predicted genes was performed using the Comprehensive Antibiotic Resistance Database (CARD), the Carbohydrate-Active Enzymes Database (CAZy), the Database of Fungal Virulence Factors (DFVF), Gene Ontology (GO) analysis, Kyoto Encyclopedia of Genes and Genomes (KEGG) analysis, the Evolutionary Genealogy of Genes: Non-supervised Orthologous Groups (eggNOG) database, the Non-Redundant (NR) protein database, Cytochrome P450 (CYP) analysis, the Pathogen–Host Interactions database (PHI), secondary metabolite analysis (antiSMASH, https://antismash.secondarymetabolites.org), and the Transporter Classification Database (TCDB) [30,31,32].

## 3. Results

### 3.1. Disease Symptoms

**Figure 1:**
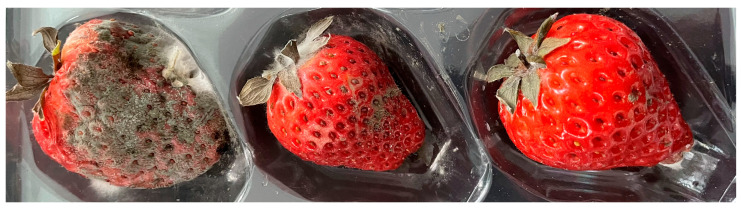
Typical symptoms of postharvest fruit rot in collected strawberry fruit.

Postharvest fruit rot was observed in 20 kg of strawberry (cv. Hongyan) fruit collected in Tai’an, China, with an incidence of approximately 30%. Initial symptoms appeared as light brown lesions, which progressively darkened and expanded, ultimately resulting in whole-fruit decay accompanied by grey and turquoise fungal growth (Figure 1).

### 3.2. Fungal Isolation

**Figure 2:**
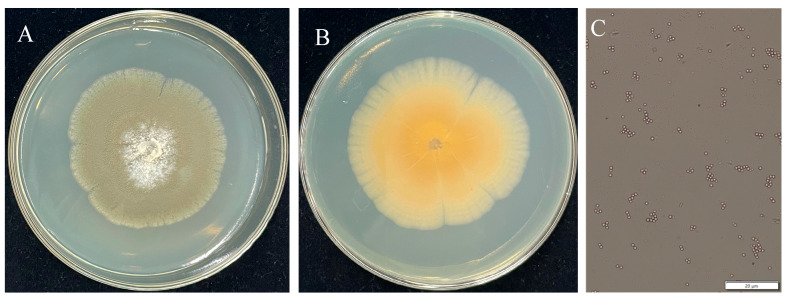
Front (**A**) and back (**B**) views of fungal CM-RB5 colonies isolated from infected strawberry fruit, and conidial morphology (**C**) of CM-RB5.

Finally, three isolated fungi were obtained through single-spore culture on PDA. A representative fungal isolate, designated CM-RB5, was isolated and cultured on PDA plates for 7 days (Figure 2A,B). Colonies were green, initially white, and guttulate. Conidia were smooth and pale green, with a subglobose to globose shape, measuring 1.2–2.4 × 1.5–2.7 µm (*n* = 50) (Figure 2C). These morphological characteristics were consistent with those of *Penicillium* species [33].

### 3.3. Molecular Identification

**Figure 3:**
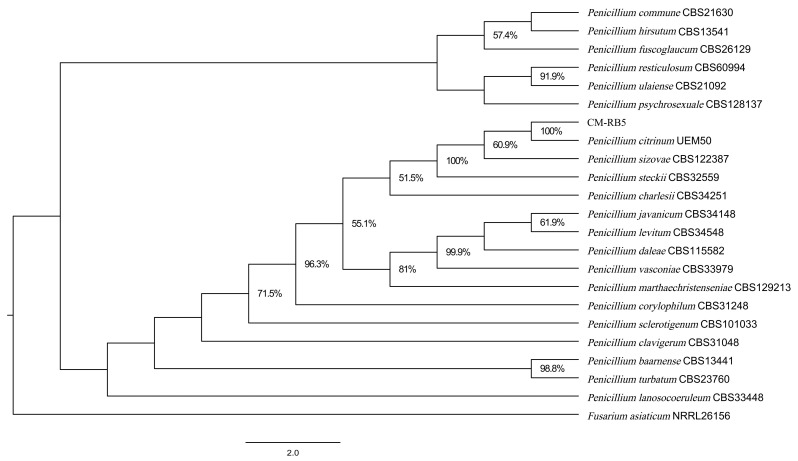
Phylogenetic tree of isolate CM-RB5 based on rDNA-ITS sequences.

The ITS sequence of isolate CM-RB5 ITS (accession no. PZ225631) exhibited 100% identity (503/503 nt) with *Penicillium citrinum* (OP329192). A phylogenetic tree (Figure 3) was constructed from a single sequence of ITS genes and showed that CM-RB5 was clustered with *P. citrinum*.

### 3.4. Pathogenicity Tests

**Figure 4:**
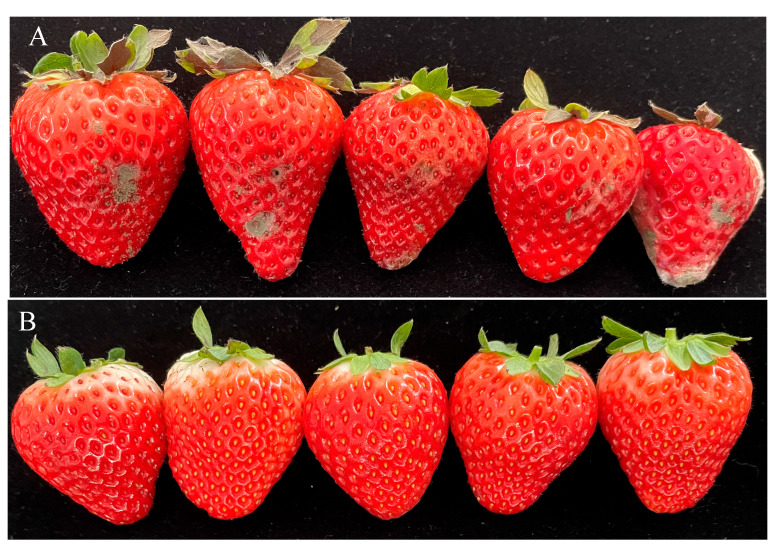
Symptoms of strawberry fruit following inoculation with CM-RB5 (**A**) and sterile water (**B**).

All five strawberry fruits inoculated with conidial suspension of CM-RB5 developed fruit rot symptoms, whereas no symptoms were observed in the control fruits inoculated with sterile distilled water (Figure 4). The pathogen was successfully re-isolated from the inoculated diseased fruit and re-identified as *P. citrinum*, thus fulfilling Koch’s postulates.

### 3.5. Genomic Analysis

**Figure 5:**
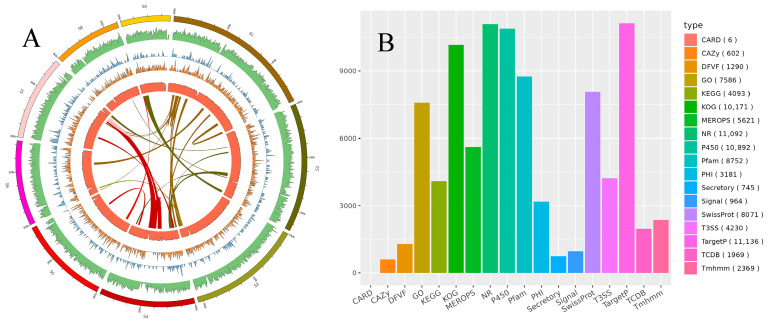
Genomic circle map of CM-RB5 (**A**) and statistical overview of protein-coding gene functional annotation (**B**).

The assembled genome of *Penicillium citrinum* CM-RB5 (Genome submission SUB16090369) had a total length of 32,053,718 bp, with a GC content of 46.41% (Figure 5A). The copy numbers of ncRNA, 5S rRNA, 5.8S rRNA, 18S rRNA, 28S rRNA, and tRNA were found to be 42, 32, 3, 3, 3, and 161, respectively, with total lengths of 5581, 3711, 394, 3594, 9418, and 13,645 bp, respectively.

Functional annotation of protein-coding genes was performed using multiple databases, including CARD (6), CAZy (602), DFVF (1290), GO (7586), KEGG (4093), KOG (10,171), MEROPS (5621), NR (11,092), P450 (10,892), Pfam (8752), PHI (3181), Secretory (745), Signal (964), SwissProt (8071), T3SS (4230), TargetP (11,136), TCDB (1969), and TMHMM (2369) (Figure 5B).

CARD analysis identified five antibiotic resistance genes and one antibiotic target, but no antibiotic biosynthesis genes, accounting for 0.054% of annotated genes (Table 1). The five genes in the “Antibiotic Resistance” category, scaffold1.t1497, scaffold1.t1543, scaffold4.t788, scaffold5.t519, and scaffold6.t97, were closely related to APH(3″)-Ib Curated, Catalase-peroxidase-peroxynitritase T KatG, thymidylate synthase, elongation factor Tu, and Monooxygenase EthA, respectively. The scaffold4.t828 gene in the “Antibiotic Target” category was closely related to translation elongation factor G.

**Figure 6:**
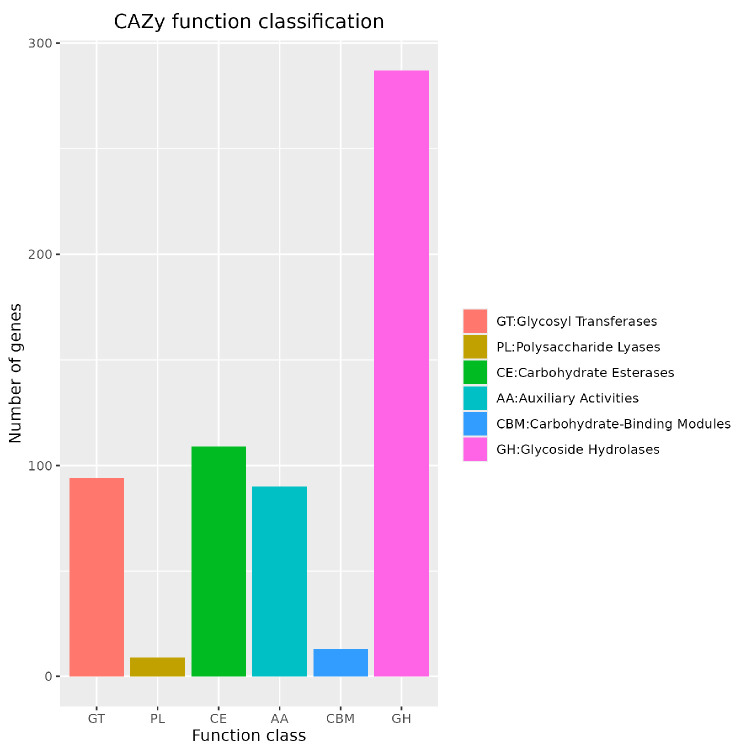
CAZy function classification profile.

CAZy database analysis (Figure 6) revealed 94 glycosyl transferases (GTs), 9 polysaccharide lyases (PLs), 109 carbohydrate esterases (CEs), 90 auxiliary activity enzymes (AAs), 13 carbohydrate-binding modules (CBMs), and 283 glycoside hydrolases (GHs). Notably, 15 polygalacturonase genes (scaffold1.t1039, scaffold1.t1358, scaffold1.t522, scaffold1.t609, scaffold1.t687, scaffold3.t649, scaffold5.t763, scaffold6.t100, scaffold6.t1009, scaffold6.t1109, scaffold7.t413, scaffold7.t689, scaffold8.t126, scaffold8.t340, and scaffold8.t469) were identified, representing typical virulence factors in *Penicillium* species (Table 2).

Two genes encoding ATP-binding cassette (ABC) and major facilitator superfamily (MFS) transporters were detected (48th and 72nd hits), which likely contribute to detoxification by exporting key phytoalexins from fungal cells (Table S1).

**Figure 7:**
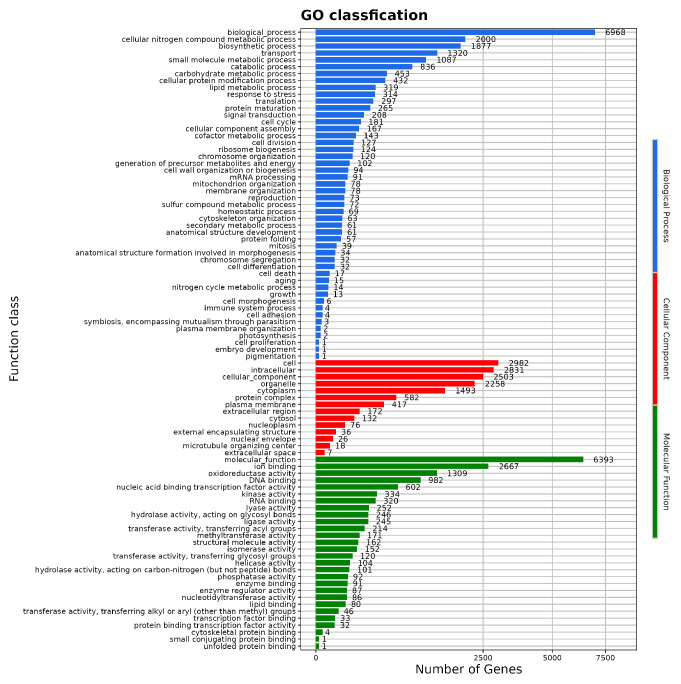
GO function classification profile.

GO analysis indicated that *P. citrinum* CM-RB5 participates in biological processes, molecular functions, and cellular components (Figure 7). A total of 38 genes were associated with toxin-related processes, including “toxin biosynthetic process (GO:0009403)”, “toxin metabolic process (GO:0009404)”, “aflatoxin biosynthetic process (GO:0045122)”, “aflatoxin metabolic process (GO:0046222)”, “mycotoxin biosynthetic process (GO:0043386)”, and “mycotoxin metabolic process (GO:0043385)” (Table S2).

**Figure 8:**
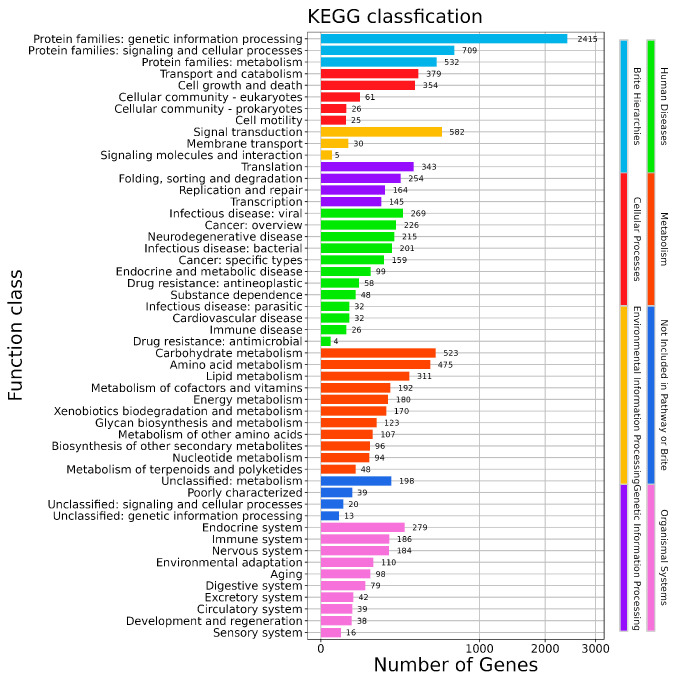
KEGG function classification profile.

KEGG analysis (Figure 8) annotated 2415 genes to “Protein families: genetic information processing” within Brite Hierarchies, 379 to “Transport and catabolism” under “Cellular Processes”, 582 to “Signal transduction” under “Environmental Information Processing”, 343 to “Translation” under “Genetic Information Processing”, 523 to “Carbohydrate Metabolism” under “Metabolism”, 198 to “Unclassified: metabolism” in “Unclassified: metabolism”, and 279 to “Endocrine system” under “Organismal Systems”.

**Figure 9:**
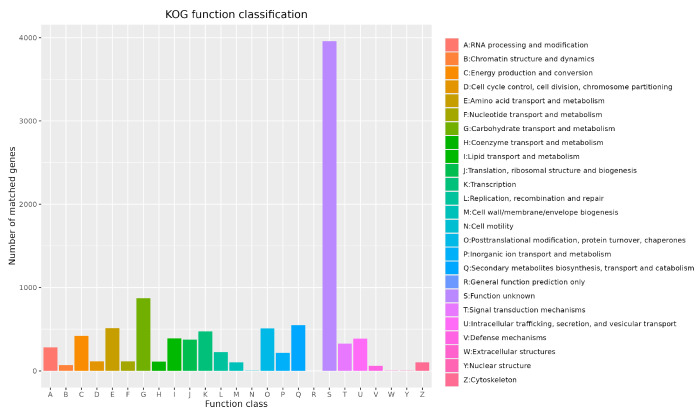
eggNOG function classification profile.

The largest category comprised 3960 genes of unknown function, accounting for 38.93% of the total, indicating that the functional annotation of *P. citrinum* CM-RB5 remains incomplete and requires further investigation (Figure 9). Notably, scaffold1.t1376 and scaffold6.t358 of *P. citrinum* CM-RB5 are involved in the production of ochratoxin and citrinin (Table 3).

**Figure 10:**
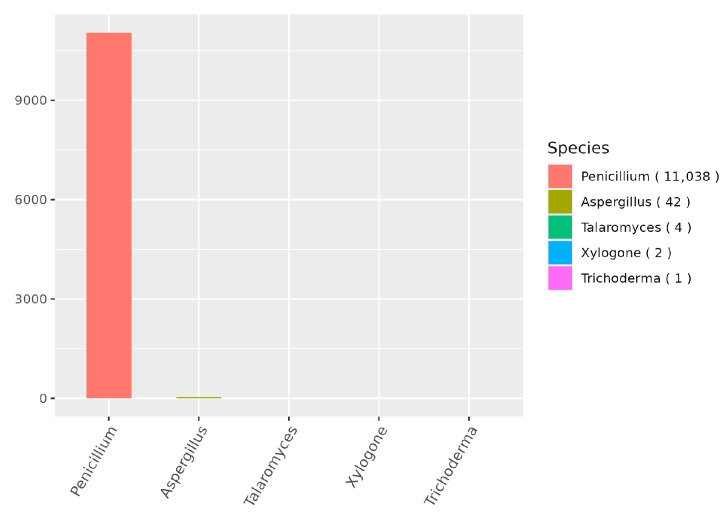
NR function classification profile.

NR analysis (Figure 10) showed that strain CM-RB5 shared the highest sequence similarity (99.56%) with members of the *Penicillium* genus. Additionally, protein-coding gene sequences exhibited similarity to proteins from *Aspergillus*, *Talaromyces*, *Xylogone*, and *Trichoderma*, with sequence lengths of 42, 4, 2, and 1 amino acids, respectively.

**Figure 11:**
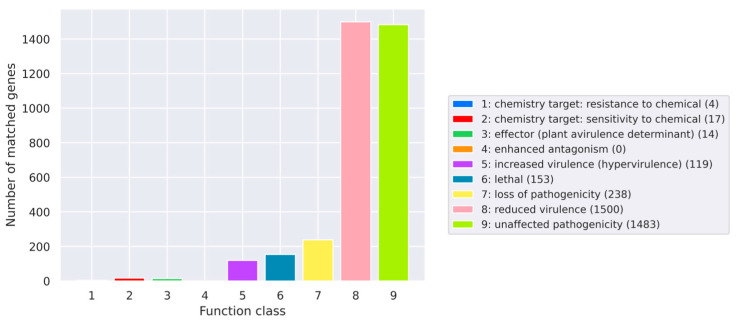
PHI function classification profile.

PHI-base analysis revealed 238 genes associated with loss of pathogenicity, 1483 with no effect on pathogenicity, 1500 with reduced virulence, and 119 with increased pathogenicity (hypervirulence) (Figure 11).

**Figure 12:**
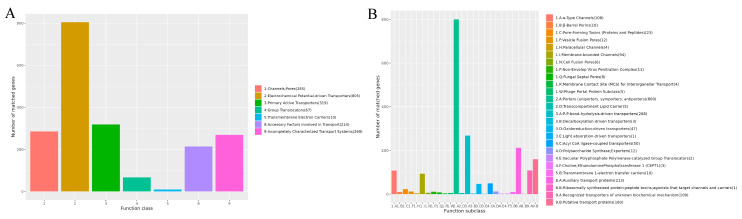
TCDB annotation species statistics chart. (**A**) Primary classification; (**B**) Secondary classification.

TCDB analysis identified 805 genes in the “Electrochemical potential-driven transporters” category within the primary classification (Figure 12A) and 800 genes in the “Porters (uniporters, symporters, antiporters)” category within the secondary classification (Figure 12B). These annotations indicate that the corresponding genes are closely associated with energy supply, self-metabolism, and toxin secretion in *P. citrinum* CM-RB5.

## 4. Discussion

*Penicillium* is a globally distributed and diverse genus that plays an important role in the decomposition of organic matter and is a major cause of spoilage in the food industry [37,38,39,40]. Many species act as postharvest pathogens, causing destructive rots in food crops; however, species identification within *Penicillium* remains challenging and could be further elaborated (e.g., morphological similarity, genetic complexity) [37,38,39,40]. Among these, *Penicillium citrinum* is a filamentous fungus widely distributed worldwide that can infect a range of hosts, including fruits such as mandarins [41], as well as *Dictyophora rubrovolvata* [33]. In this study, *P. citrinum* CM-RB5 was isolated from infected strawberry fruit and identified based on spore morphology and molecular analyses. This isolate was confirmed to cause postharvest rot in strawberry fruit. In China, more than 170 *Penicillium* species have been recorded, of which 91 were originally described from this country [42]. To the best of our knowledge, this is the first report of *P. citrinum* causing postharvest strawberry fruit rot worldwide. Further studies should focus on disease management strategies, including biological, chemical, and physical control approaches, to prevent the localized occurrence [41,43,44].

*Penicillium citrinum* often disrupts plant and fruit tissue structure by secreting cell wall-degrading enzymes. During infection, it degrades cell wall components, causing the fruits to soften and rot. Postharvest diseases caused by *P. citrinum* not only lead to substantial economic losses but also severely restrict the development of the fruit industry. Genomic analyses have been conducted for several *Penicillium* species, including *P. exsudans* [45], *P. commune* [46], and *P. turbatum* [47]. The availability of genomic data enhances the resolution of intraspecies comparisons and advances our understanding of evolutionary biology [46]. In this study, we present several lines of evidence (Figure 2, Figure 3 and Figure 4) supporting the identification of the isolated strain as the understudied *P. citrinum*, along with its high-quality genome sequence. This resource provides a platform for identifying pathways and gene clusters encoding proteins involved in the biosynthesis of small molecules of interest in *P. citrinum*.

CAZy database analysis revealed that *P. citrinum* CM-RB5 has 109 CEs, which catalyse the hydrolysis of ester bonds in carbohydrates, facilitating the decomposition of complex sugars such as cellulose and lignin and providing essential carbon sources for fungal growth [31]. Moreover, *P. citrinum* CM-RB5 contains nine PLs, which catalyse polysaccharide cleavage, specifically breaking down complex sugars such as pectin and starch into accessible sugars [31]. *P. citrinum* CM-RB5 possesses 283 GHs, which hydrolyse glycosidic bonds, breaking down polysaccharides such as cellulose, starch, and pectin to supply the fungus with necessary sugars [31]. Furthermore, 94 GTs were detected; these enzymes play a crucial role in carbohydrate synthesis by catalysing the transfer of carbohydrate units to form new glycoside chains [31]. These processes are closely associated with the infection of strawberry fruits by *P. citrinum* CM-RB5. *P. citrinum* CM-RB5 encodes multiple cytochrome P450 enzymes, including cytochrome P450 monooxygenase, cytochrome P450 phenylacetate hydroxylase, and benzoate 4-monooxygenase cytochrome P450. These CYP-related isoenzymes are involved in cellular detoxification, xenobiotic degradation, and the biosynthesis of secondary metabolites during host–pathogen interactions. Accordingly, research on fungal CYP has expanded rapidly and constitutes an important area within biology and ecology.

*Penicillium* species are necrotrophic pathogens of fruits that penetrate host tissues through wounds and subsequently colonise beyond the initial infection site using a series of virulence factors, which notably include the production of polygalacturonase [48]. Several *Penicillium* species, with varying levels of virulence, have been widely used as model systems to investigate genes, pathways, and metabolites involved in fungal-mediated fruit decay (e.g., in apple) [46]. In the present study, 15 polygalacturonase-encoding genes were identified in *P. citrinum* CM-RB5, which are closely associated with its pathogenicity. In addition, secondary metabolites, or natural products, are an invaluable resource for biotechnological applications, but also raise concern due to their potential impacts on health [46]. Furthermore, *P. citrinum* produces ochratoxin and citrinin inferred from genomic data, two toxins that not only damage plant cells but also contaminate agricultural products, posing a serious threat to food safety [49,50,51]. Future management (e.g., storage conditions, biological control, toxin mitigation) of postharvest strawberry fruit rot caused by *P. citrinum* CM-RB5 should prioritise reducing infection incidence and mitigating the harmful effects caused by these toxins.

## 5. Conclusions

*P. citrinum* CM-RB5, isolated from infected strawberry fruit and identified based on conidial morphology and molecular analysis, was confirmed to cause postharvest strawberry fruit rot. Our study represents the first report of *P. citrinum* as a causal agent of postharvest fruit rot in China. Genome assembly revealed that *P. citrinum* CM-RB5 has a total length of 32,053,718 bp and a GC content of 46.41%. Functional annotation of protein-coding genes identified matches across multiple databases, including CARD (6), CAZy (602), DFVF (1290), GO (7586), KEGG (4093), KOG (10,171), MEROPS (5621), NR (11,092), P450 (10,892), Pfam (8752), PHI (3181), Secretory (745), Signal (964), SwissProt (8071), T3SS (4230), TargetP (11,136), TCDB (1969), and TMHMM (2369) analysis. Furthermore, *P. citrinum* CM-RB5 can produce ochratoxin and citrinin, which constitute a serious threat for animals and humans. Future studies should focus on developing effective control measures to manage postharvest strawberry fruit rot caused by *P. citrinum*.

## Figures and Tables

**Table 1:** Antibiotic resistance analysis statistics in CARD.

Property	Number of Genes	Percentage (%)	Query_Name
Antibiotic Resistance	5	0.045	scaffold1.t1497 scaffold1.t1543 scaffold4.t788 scaffold5.t519 scaffold6.t97
Antibiotic Target	1	0.009	scaffold4.t828
Antibiotic Biosynthesis	0	0	-

**Table 2:** Polygalacturonase description in CAZy.

Gene	Family	Evalue
scaffold1.t1039	GH28	9.5 × 10^−68^
scaffold1.t1358	GH28	8.3 × 10^−72^
scaffold1.t522	GH28	3.8 × 10^−74^
scaffold1.t609	GH28	1.7 × 10^−66^
scaffold1.t687	GH28	2.4 × 10^−65^
scaffold3.t649	GH28	4.4 × 10^−71^
scaffold5.t763	GH28	2.5 × 10^−65^
scaffold6.t100	GH28	2.5 × 10^−72^
scaffold6.t1009	GH28	8.9 × 10^−75^
scaffold6.t1109	GH28	4.2 × 10^−74^
scaffold7.t413	GH28	8.6 × 10^−68^
scaffold7.t689	GH28	7.5 × 10^−67^
scaffold8.t126	GH28	2.3 × 10^−52^
scaffold8.t340	GH28	1.1 × 10^−49^
scaffold8.t469	GH28	1.7 × 10^−72^

**Table 3:** Analysis of ochratoxin and citrinin in eggNOG.

Gene	Length	Function
scaffold6.t358	789	Citrinin biosynthesis oxydoreductase CtnB
scaffold7.t994	1074	Amidohydrolase family, ochratoxinase

## Data Availability

The original contributions presented in this study are included in the article/Supplementary Materials. Further inquiries can be directed to the corresponding author.

## Supplementary Materials

The following supporting information can be downloaded at: https://www.mdpi.com/article/10.3390/jof12040288/s1, Table S1. Analysis of ABC and MFS transporters in DFVF. Table S2. Toxin-related processes in GO.
