# Supplementary material for: Emergence of Postharvest Strawberry Fruit Rot Caused by Penicillium citrinum in China and Its Whole-Genome Sequencing
Source: J Fungi (Basel). 2026 Apr 17;12(4):288. doi: 10.3390/jof12040288 (PMC13117804; doi:10.3390/jof12040288)
Supplement: Supplementary file 1 [file jof-12-00288-s001.zip › jof-4266269-supplementary.pdf]

Table S1. Analysis of ABC and MFS transporters in DFVF

Function	Query_name	Hit_length	Hit_start	Hit_end	Aln_length	Identity	Evalue
	scaffold1.t32	1439	79	1438	1375	49.09	0
	scaffold1.t156	1321	367	622	258	33.33	1.06e-29
	scaffold1.t274	1501	41	1478	1450	63.72	0
	scaffold1.t494	1606	444	1603	1180	27.54	3.05e-117
	scaffold1.t579	1501	223	437	215	61.86	2.53e-80
	scaffold1.t580	1501	71	188	119	44.54	3.43e-28
	scaffold1.t716	1562	34	1556	1533	61.06	0
	scaffold1.t1038	1321	22	1321	1311	41.95	0
	scaffold1.t1123	1439	59	1439	1385	60.07	0
	scaffold1.t1551	1439	58	1439	1388	64.63	0
	scaffold1.t1560	1606	232	1598	1420	25.99	9.08e-115
	scaffold1.t1737	1321	26	1319	1298	33.59	1.39e-227
	scaffold2.t50	1635	126	1533	1430	53.15	0
	scaffold2.t508	1491	865	1424	583	26.42	3.98e-47
	scaffold2.t869	1606	1469	1591	128	40.63	1.59e-17
	scaffold2.t959	1439	54	1439	1387	71.95	0
	scaffold2.t1177	1321	18	1320	1389	28.87	1.95e-137
	scaffold2.t1210	1321	29	1320	1378	30.33	6.91e-162
	scaffold2.t1539	1484	839	1302	492	29.47	1.53e-48
ABC transporter superfamily.	scaffold3.t165	1484	39	1484	1462	61.56	0
	scaffold3.t700	1321	351	625	277	41.52	1.27e-46
	scaffold3.t949	1439	187	1426	1255	43.59	0
	scaffold3.t1349	1439	57	1438	1389	64.07	0
	scaffold3.t1357	1321	32	1321	1318	36.12	2.33e-235
	scaffold4.t49	1501	77	1481	1407	60.56	0
	scaffold4.t855	1606	472	1603	1190	32.27	8.56e-148
	scaffold4.t936	1321	188	1318	1244	25	7.61e-69
	scaffold4.t987	1606	426	1603	1224	32.11	5.49e-158
	scaffold4.t1030	1606	1295	1598	309	35.28	1.22e-42
	scaffold4.t1059	1562	215	457	249	31.33	1.27e-27
	scaffold4.t1123	1606	261	1580	1382	22.65	6.53e-78
	scaffold5.t189	1321	363	631	272	41.18	2.22e-47
	scaffold5.t417	1501	36	1479	1459	63.19	0
	scaffold5.t441	1606	1108	1600	496	29.23	6.06e-55
	scaffold5.t442	1635	367	1532	1179	59.88	0
	scaffold5.t443	1635	102	362	265	53.21	1.12e-84
	scaffold5.t588	1321	34	1320	1373	29.72	1.29e-147
	scaffold5.t815	1606	284	1598	1336	27.02	8.96e-113
	scaffold5.t908	1606	283	1591	1356	25.96	7.17e-107
	scaffold6.t110	1562	148	1505	1378	44.05	0
	scaffold6.t975	1501	23	1482	1468	60.01	0

	scaffold7.t362	1606	426	1606	1230	31.38	3.02e-164
	scaffold7.t532	1606	59	1606	1584	38.70	1.22e-310
	scaffold7.t1023	1439	79	1438	1373	48.07	0
	scaffold7.t1047	1439	59	1439	1382	71.49	0
	scaffold8.t647	1321	26	1310	1357	29.11	1.45e-155
	scaffold8.t725	1635	134	1525	1429	62.21	0
	scaffold9.t109	1606	408	1578	1212	27.31	1.37e-100
major facilitator superfamily	scaffold1.t58	748	39	496	488	32.79	3.59e-68
	scaffold1.t100	615	109	550	462	29.44	1.69e-39
	scaffold1.t374	615	101	544	472	23.73	7.96e-20
	scaffold1.t380	615	150	550	414	27.78	1.08e-28
	scaffold1.t381	615	135	548	439	23.92	4.97e-26
	scaffold1.t554	748	43	495	468	40.82	4.85e-116
	scaffold1.t567	615	151	550	406	22.91	4.05e-22
	scaffold1.t646	615	69	581	515	62.52	1.90e-229
	scaffold1.t824	748	42	496	486	30.86	1.86e-59
	scaffold1.t1062	615	103	548	482	26.76	6.67e-44
	scaffold1.t1072	615	108	550	463	22.89	2.50e-13
	scaffold1.t1279	615	98	552	480	21.46	1.01e-14
	scaffold1.t1353	615	103	548	479	25.26	1.54e-27
	scaffold1.t1677	748	43	495	461	45.77	2.53e-123
	scaffold2.t434	748	39	496	477	25.58	5.39e-34
	scaffold2.t439	615	103	563	469	28.14	2.88e-59
	scaffold2.t594	615	103	552	460	28.04	1.14e-53
	scaffold2.t765	748	43	493	463	30.45	5.06e-51
	scaffold2.t782	748	39	496	473	30.66	3.11e-53
	scaffold2.t905	615	150	555	432	26.16	1.15e-22
	scaffold2.t954	615	104	552	483	26.29	1.17e-36
	scaffold2.t1150	615	132	550	441	26.98	4.56e-28
	scaffold2.t1157	748	39	492	465	26.67	1.81e-34
	scaffold2.t1616	748	42	496	499	30.86	4.73e-61
	scaffold2.t1622	615	113	550	464	28.02	3.02e-36
	scaffold3.t708	615	150	550	413	24.21	1.60e-22
	scaffold3.t815	615	98	549	490	23.47	5.35e-22
	scaffold3.t1035	615	143	548	452	24.34	5.23e-22
	scaffold3.t1405	615	103	561	473	29.39	2.00e-58
	scaffold3.t1437	615	150	552	418	28.23	8.52e-37
	scaffold4.t24	748	42	496	476	30.88	1.85e-47
	scaffold4.t76	615	167	297	139	30.94	4.97e-08
	scaffold4.t115	748	27	496	484	28.10	1.21e-54
	scaffold4.t124	615	99	550	467	24.84	1.27e-32
	scaffold4.t156	748	36	492	481	30.35	1.81e-41
	scaffold4.t239	748	32	509	485	42.68	5.98e-135
	scaffold4.t280	748	43	496	485	30.31	6.40e-56

scaffold4.t376	615	113	545	446	24.22	1.75e-21
scaffold4.t387	615	112	382	276	23.19	9.72e-21
scaffold4.t970	615	109	553	487	28.75	1.56e-44
scaffold4.t1011	615	93	574	521	24.76	1.28e-32
scaffold5.t347	615	104	548	477	26.62	5.16e-32
scaffold5.t689	748	39	508	483	37.89	1.53e-92
scaffold5.t827	748	23	510	492	42.28	1.13e-125
scaffold5.t840	615	151	550	422	23.23	1.92e-13
scaffold5.t1024	748	27	496	488	32.58	7.99e-58
scaffold5.t1149	748	71	497	441	35.15	1.16e-74
scaffold6.t15	748	42	493	483	31.68	6.82e-71
scaffold6.t276	615	101	549	484	25.41	8.95e-31
scaffold6.t352	615	143	550	424	26.89	3.74e-30
scaffold6.t430	748	37	496	471	27.81	2.11e-51
scaffold6.t807	615	106	554	458	27.51	3.18e-51
scaffold6.t934	748	39	496	499	30.86	1.93e-62
scaffold6.t973	615	150	548	416	27.64	1.81e-28
scaffold7.t348	615	110	557	472	25.42	9.63e-23
scaffold7.t349	615	101	548	504	30.16	8.05e-53
scaffold7.t579	615	136	548	433	30.25	2.97e-30
scaffold7.t624	615	111	550	464	27.16	1.58e-34
scaffold7.t761	615	69	575	515	61.75	1.10e-221
scaffold7.t773	748	44	496	459	29.85	2.39e-54
scaffold7.t986	615	149	550	419	22.91	1.87e-17
scaffold8.t20	748	39	498	473	33.83	1.43e-75
scaffold8.t218	748	39	520	504	27.98	1.02e-45
scaffold8.t559	615	84	551	498	28.11	1.40e-35
scaffold8.t727	615	91	561	532	27.82	2.34e-51
scaffold8.t776	615	143	608	488	28.48	1.38e-45
scaffold8.t838	615	103	559	487	25.87	1.52e-29
scaffold9.t284	748	32	496	495	33.94	4.79e-69
scaffold9.t481	748	85	497	418	30.38	2.42e-39
scaffold9.t490	615	101	561	482	25.31	3.58e-31
scaffold9.t513	615	145	550	415	27.71	1.30e-30
scaffold9.t616	615	98	549	479	25.05	1.90e-28

Table S2. Toxin-related processes in GO.

protein_id	GO_number	GO_function	GO_ontologies
scaffold1.t257	GO:0009403	toxin biosynthetic process	Biological Process
scaffold1.t257	GO:0009404	toxin metabolic process	Biological Process
scaffold1.t756	GO:0009403	toxin biosynthetic process	Biological Process
scaffold1.t756	GO:0009404	toxin metabolic process	Biological Process
scaffold1.t756	GO:0046222	aflatoxin metabolic process	Biological Process

scaffold1.t756	GO:0043385	mycotoxin metabolic process	Biological Process
scaffold1.t756	GO:0043386	mycotoxin biosynthetic process	Biological Process
scaffold1.t756	GO:0045122	aflatoxin biosynthetic process	Biological Process
scaffold1.t771	GO:0009403	toxin biosynthetic process	Biological Process
scaffold1.t771	GO:0009404	toxin metabolic process	Biological Process
scaffold2.t70	GO:0009404	toxin metabolic process	Biological Process
scaffold2.t70	GO:0009403	toxin biosynthetic process	Biological Process
scaffold2.t362	GO:0009403	toxin biosynthetic process	Biological Process
scaffold2.t362	GO:0009404	toxin metabolic process	Biological Process
scaffold4.t73	GO:0046222	aflatoxin metabolic process	Biological Process
scaffold4.t73	GO:0043385	mycotoxin metabolic process	Biological Process
scaffold4.t73	GO:0043386	mycotoxin biosynthetic process	Biological Process
scaffold4.t73	GO:0009403	toxin biosynthetic process	Biological Process
scaffold4.t73	GO:0009404	toxin metabolic process	Biological Process
scaffold4.t73	GO:0045122	aflatoxin biosynthetic process	Biological Process
scaffold4.t74	GO:0046222	aflatoxin metabolic process	Biological Process
scaffold4.t74	GO:0043385	mycotoxin metabolic process	Biological Process
scaffold4.t74	GO:0043386	mycotoxin biosynthetic process	Biological Process
scaffold4.t74	GO:0009403	toxin biosynthetic process	Biological Process
scaffold4.t74	GO:0009404	toxin metabolic process	Biological Process
scaffold4.t74	GO:0045122	aflatoxin biosynthetic process	Biological Process
scaffold4.t853	GO:0009403	toxin biosynthetic process	Biological Process
scaffold4.t853	GO:0009404	toxin metabolic process	Biological Process
scaffold7.t333	GO:0046222	aflatoxin metabolic process	Biological Process
scaffold7.t333	GO:0043385	mycotoxin metabolic process	Biological Process
scaffold7.t333	GO:0043386	mycotoxin biosynthetic process	Biological Process
scaffold7.t333	GO:0009403	toxin biosynthetic process	Biological Process
scaffold7.t333	GO:0009404	toxin metabolic process	Biological Process
scaffold7.t333	GO:0045122	aflatoxin biosynthetic process	Biological Process
scaffold7.t1044	GO:0009403	toxin biosynthetic process	Biological Process
scaffold7.t1044	GO:0009404	toxin metabolic process	Biological Process
scaffold9.t228	GO:0009403	toxin biosynthetic process	Biological Process
scaffold9.t228	GO:0009404	toxin metabolic process	Biological Process
